# Minimization of the Influence of Shear-Induced Particle Migration in Determining the Rheological Characteristics of Self-Compacting Mortars and Concretes

**DOI:** 10.3390/ma13071542

**Published:** 2020-03-27

**Authors:** Christian Baumert, Harald Garrecht

**Affiliations:** Institute of Construction Materials, University of Stuttgart, 70569 Stuttgart, Germany; harald.garrecht@iwb.uni-stuttgart.de

**Keywords:** shear-induced particle migration, rheology, active pre-shear, KNIELE KKM-RT, rheometer, Reiner-Riwlin, modified Bingham

## Abstract

Determining the exact rheological properties of cementitious materials in fundamental units is a crucial step in concrete science. It is undisputed that before measuring rheological properties in concrete rheometers, it is necessary to pre-shear the fresh mortar or concrete. Due to the migration of the coarse particles into areas with lower shear stress, however, segregation takes place. An experimental set-up was developed to determine the effects on the measured values of the concrete rheometer ICAR. This allows the active homogenization (pre-shearing) of the material before each change of speed. In the tests higher raw values (macroscopic data) could be measured. This clearly influences the calculated rheological Bingham parameters and modified Bingham parameters for a self-compacting concrete (SCC) with a maximum grain size of 16 mm. Therefore, the homogeneity of the material, a main hypothesis of rheological measurements, does not seem to be fulfilled with the coaxial rheometer used. The process of the indispensable pre-shearing therefore requires more attention in the future so that measurement errors can be minimized. Especially in numerical simulation, suitable rheological models and the realistic determination of parameters are crucial. Since the shear-induced particle migration is largely dependent on the maximum grain size, an ultra-high performance concrete (UHPC) with a maximum particle size of only 0.5 mm was also investigated in the laboratory mixer KNIELE KKM-RT. The integrated rheometer enables also the active homogenization of the fresh concrete during pre-shearing but without the danger of over-mixing, as it is the case for the experimental ICAR setup. This article proves that relevant differences can also be identified for such a material.

## 1. Introduction

Self-compacting concretes, compacting and de-aerating under the influence of gravity, offer advantages during placing and are known for excellent quality of the concrete surface [1]. However, these concretes only perform in the intended way if the flow properties are within a permissible range [2]. Therefore, the importance of measuring the rheological properties of fresh concrete is increasing. Especially when carrying out numerical simulations, the realistic values, and their development over time, are absolutely necessary. However, these values are also needed for other considerations. In particular, the pumping of concrete, the filling of formwork, shotcrete and 3D-printing. 

The Bingham model (Equation (1)) is the most widely used rheological model for traditional vibrated concrete. As usual with viscoplastic materials, the yield stress *τ*_0_ must be overcome to initialize the flow. Once the yield stress *τ*_0_ is passed, there is a linear relationship between the shear stress *τ* and the shear rate $\dot{\gamma }$, which is called plastic viscosity (*μ*).

(1) $$\tau =\tau_{0}+\mu \times \dot{\gamma }$$

These parameters can be experimentally determined using a rheometer. To eliminate the time-dependency of mixtures, experimental tests will be done at a defined time. Furthermore, shear stress and, shear rate and plastic viscosity are considered constant for a given mixture. Moreover, an independence of these physical parameters of possible changes in the distribution of the granular phase is assumed. A plot of shear stress *τ* versus shear rate $\dot{\gamma }$ is defined as a flow curve. For many concretes, however, flow curves are determined that cannot be adequately described by the linear Bingham model. For a long time, the Herschel–Bulkley model (Equation (2)) was therefore favoured for shear-thinning (pseudoplastic) or shear-thickening (rheopex) behaviour.

(2) $$\tau =\tau_{0}+K\times {\dot{\gamma }}^{n}$$

In the meantime, it has been shown that the Herschel-Bulkley model is not the best to describe non-linear behaviour [3]. Furthermore [4] have shown that the Herschel-Bulkley model always gives the lowest value for yield stress in the case of shear thinning and the highest value in the case of shear thickening. The yield stress calculated by the modified Bingham model is always between the Herschel-Bulkley and Bingham yield stresses and therefore the modified Bingham model could provide a more accurate estimation of the yield stress.

(3) $$\tau =\tau_{0}+\mu \times \dot{\gamma }+c\times {\dot{\gamma }}^{2}$$

In order to transform the raw data or macroscopic data speed and torque of the rheometer into the absolute rheological units, the equation according to Reiner–Riwlin (Equation (4)), is used.

(4) $$T=\frac{4\pi \ln\frac{R_{s}}{R_{i}}}{\left(\frac{1}{R_{i}^{2}}-\frac{1}{R_{s}^{2}}\right)}\times \tau_{0}+\frac{8h\pi^{2}}{\left(\frac{1}{R_{i}^{2}}-\frac{1}{R_{s}^{2}}\right)}\times \mu \times N$$

with:

*T*  torque measured in coaxial cylinders rheometer (Nm);
*R_s_*  boundary between sheared and unsheared material (m);
*R_i_*  inner cylinder radius of coaxial cylinders rheometer (m);
*h*  high of inner cylinder (m);
*N*  rotational velocity measured in coaxial cylinders rheometer (rps).

It is necessary to check whether the material is sheared in the entire gap for each speed-torque point. If not, iterations have to be performed, e.g., by using the Excel Solver. For the modified Bingham model a solution has been developed by [4].

(5) $$T=\frac{4\pi \ln\frac{R_{s}}{R_{i}}}{\left(\frac{1}{R_{i}^{2}}-\frac{1}{R_{s}^{2}}\right)}\times \tau_{0}+\frac{8h\pi^{2}}{\left(\frac{1}{R_{i}^{2}}-\frac{1}{R_{s}^{2}}\right)}\times \mu \times N+\frac{8\pi^{3}h}{\left(\frac{1}{R_{i}^{2}}-\frac{1}{R_{s}^{2}}\right)}\times \frac{\left(R_{s}+R_{i}\right)}{\left(R_{s}-R_{i}\right)}\times c\times N^{2}$$

Already, the determination of the raw data (macroscopic data) from the rheometer for cement-based materials is not trivial. Measurement artifacts must be taken into account due to the intrinsic properties of material (thixotropy and segregation) and due to the different geometry of the rheometer in use. Therefore, the users of concrete rheometers face a dilemma. Mortar and concrete form a structure at rest, which can only be broken down by shearing the material before the actual measurement. However, this pre-shear is inevitably overlaid by shear-induced particle migration (SIPM). 

In fresh mixes of concrete particles migrate from zones with high shear intensity to zones with lower shear intensity. Areas with reduced aggregate content will show significantly lower rheological properties [2]. Since in the areas with low shear intensity the viscosity increases due to the additional particles, the SIPM is counteracted [5]. However, SIPM induces heterogeneity in the mixture during rheological measurements. [5] denotes the influence of the SIPM during the slump test and for casting of concrete as negligible. In contrast, heterogeneities in the range of 3–5% are specified for pumping and in rheometers [5]. In [6,7] the rheological properties of both concrete bulk and lubricating layer due to particle migration and the relationship between pumping pressure loss and the discharge rate in a pipeline due to particle migration was analyzed.

For a long time, the thesis was maintained that this effect is only relevant at very high shear rates [8]. However, imaging techniques have shown that even the boundary conditions that are common during pre-shear in the concrete rheometer lead to considerable vertical [9] and radial segregation [8]. As a result, the material is no longer homogeneous in composition and falsifies the measured values of the rheometer. According to [8] “it is clearly observed that the macroscopic measurements tend to underestimate the apparent viscosity of the material at any rate and to underestimate its yield stress”. [8] summarizes that “this phenomenon is most likely the main source of error in cementitious materials rheology”. [10] note that SIPM is completed approximately 20–30 s after starting the rheometer. As the ICAR Rheometer is delivered with a pre-shear time of 20 s, SIPM can be considered as completed after the end of the pre-shear time. Unfortunately, the requirement for a homogeneous material for the rheological measurements after pre-shearing is therefore not fulfilled.

Firstly, the influence of the shear induced particle migration on the measured raw data of a conventional coaxial concrete rheometer shall be determined in this article. For this purpose, the ICAR rheometer was integrated into an experimental setup that allows active homogenization of the mixture without the blade agitator of the rheometer. The measuring process can thus be divided into several individual steps in which a homogeneous mixture is always present at the start of the measurement. It can be shown in the article that the type and duration of pre-shearing massively influences the raw data. To demonstrate also the effects on the rheological models, the raw data obtained in this way are incorporated into the Bingham model and the modified Bingham model.

Secondly, rheological measurements on a fine-grained fiber-reinforced UHPC with the KNIELE KKM-RT are presented. The KKM-RT is a combination of intensive mixer and rheometer. The experimental setup for the ICAR rheometer is very time-consuming and complex, but here the processes are automated and functionally combined in one machine. In addition, there is no risk of overmixing with the KNIELE KKM-RT, as the material transport is forced even at low speed.

## 2. Materials and Methods 

**Figure 1:**
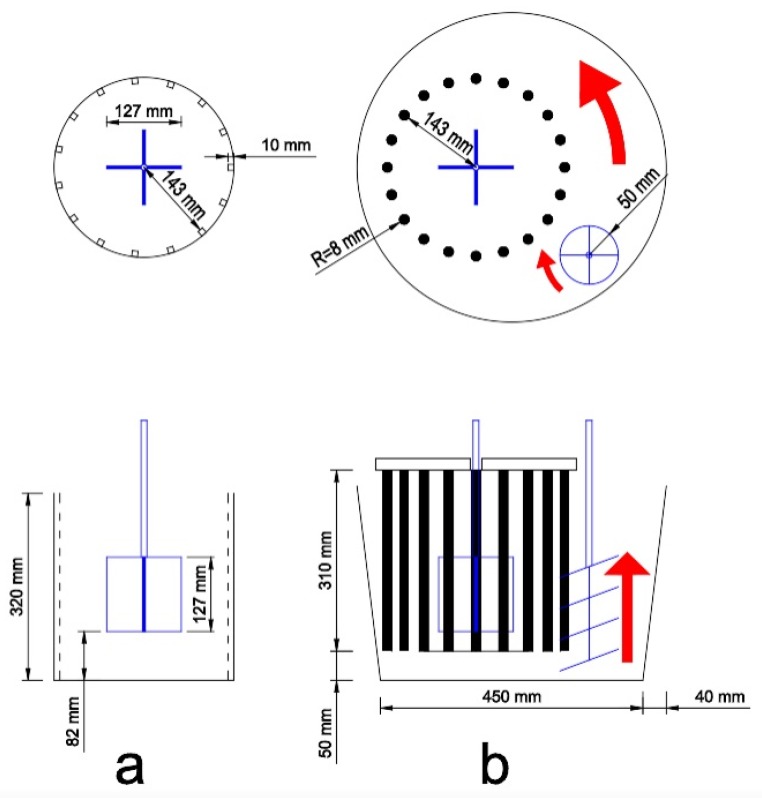
(**a**) Schematic diagram of the ICAR rheometer with the blade agitator in a container. (**b**) Schematic diagram of the experimental setup with rotating bucket and the whisk for the rheometer ICAR.

**Figure 2:**
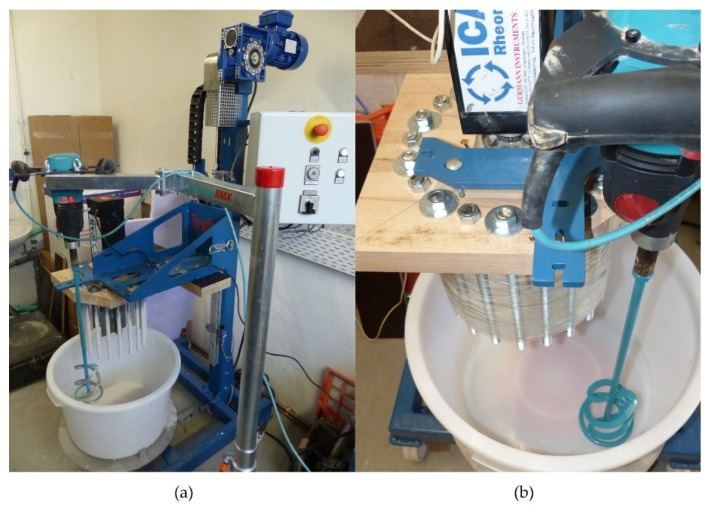
(**a**) Experimental setup for the ICAR rheometer; (**b**) the threaded rods were wrapped from the outside with several layers of adhesive tape for TEST 1.

The experimental setup for the ICAR rheometer is shown in Figure 1 and Figure 2. The individual components of the experimental setup are as follows:

- A conical bucket (Transoplast GmbH, Emmerich, Germany) with 45l content (high 330 mm; diameter 450 mm at the bottom; diameter 530 mm at the top);
- A mobile stand mixer type beba B99 (beba Technology, Essen (Oldenburg), Germany); the bucket is fixed on the base plate. If required, the container can be rotated counterclockwise by a motor with variable speed. In addition, the stand mixer has a device holder that can be moved vertically by a motor;
- A motor whisk with speed control from Collomix type XO6 attached to a Collomix mixer stand type RMX (Collomix GmbH, Gaimersheim, Germany). This mixer stand operates independent from the beba B99 and allows the motor whisk to swivel at an adjustable height in the material. The whisk with a diameter of 100 mm rotates clockwise. The axial transport upwards counteracts sedimentation. The material flow directed radially inwards counteracts the shear-induced particle migration;
- The rheometer device ICAR with blade agitator is attached to the device holder of the stand mixer beba type B99;
- The original container of the rheometer was reproduced by 20 threaded rods M16 fixed on a base plate to ensure the circulation of the material in the bucket. The base plate is attached to the device holder of the stand mixer beba type B99.

In contrast to the original solution, the pre-shearing of the mixture is therefore no longer done with the ICAR blade agitator, but with the rotating bucket and the motor whisk before performing TEST 1–TEST 5. The measuring regime in the delivery condition of the rheometer ICAR was used as reference. Firstly, the material is pre-sheared for 20 s at 0.5 revolutions per second with the ICAR blade agitator (TEST 1, TEST 2 and TEST 5). In TEST 3 and TEST 4, deviating from the reference, the duration of pre-shearing with the ICAR blade agitator was reduced from 20 s to 1 s. The data during pre-shearing with the blade agitator are not taken into account for the evaluation. Then (except TEST 4) the speed is reduced in 7 equal steps from 0.5 to 0.05 revolutions per second. In each step the speed is maintained for 5 s. The measured data in the first second after the speed changes are not taken into account to wait for the formation of a flow equilibrium in the shear gap. In TEST 4 the measurement was divided into 7 individual measurements at 0.5, 0.425, 0.35, 0.275, 0.2, 0.125 and 0.05 rev/s. Before each individual measurement, the material was thoroughly homogenized in the rotating bucket with the motorized whisk. Afterwards, the pre-shearing followed with the ICAR blade agitator for 1 s, as the software is forcing this. Afterwards, the measurement was performed at the respective speed. The data of the 7 individual measurements were then combined and evaluated. The following tests were carried out with the self-compacting concrete (Table 1). The concrete was developed on the basis of the modified Andreassen model (Equation (6)) in order to achieve sedimentation stability through a high packing density and simultaneously very good flowability [11]. In this respect, significant changes in the volume fractions of the materials would lead to an unstable system. It is therefore important to keep the volume fractions as accurate as possible in self-compacting cementitious systems. It would be possible to set the shear rate so low that shear-induced particle migration becomes insignificant. However, the shear range relevant in practice during transport and installation has to be covered and the non-linear behaviour of the material must be recorded. In this respect, the shear range selected by the manufacturer of the rheometer ICAR is relevant and has to be realistically assessed in the measurements.

(6) $$CFPD=\frac{\left(d^{q}-d_{min}^{q}\right)}{\left(D^{q}-d_{min}^{q}\right)}\times 100$$

with:

*CFPD*  cumulative (volume) percent;
*d*  particle size;
*d_min_*  minimum particle size of distribution (chosen: 0.2 µm);
*D*  maximum particle size of distribution (chosen: 16,000 µm);
*q*  distribution coefficient or exponent (chosen: 0.28).

- TEST 1: In order to test the replica of the original container by the 20 threaded rods for suitability, the threaded rods were wrapped from the outside with several layers of adhesive tape. Testing 30 min after adding water to the mix;
- TEST 2: The adhesive tape was removed from the outside of the rods. Testing 33 min after adding water to the mix;
- TEST 3: Like TEST 2, but the pre-shearing with the blade agitator was reduced to 1 s. Testing 36 min after adding water to the mix;
- TEST 4: Divided into 7 individual tests with speeds of 0.5–0.05 revolutions per second. The pre-shearing with the blade agitator at the respective speed was carried out for only 1 s each time. Prior to each individual test, the mix was homogenized with the rotating bucket and the whisk. Testing 60 min after adding water to the mix;
- TEST 5: Like TEST 2, to evaluate the rheological properties according to Test 4 and thus 70 min after adding water to the mix with the original measuring regime of the ICAR rheometer.

To test the effects of shear-induced particle migration on mortar, a fiber-reinforced UHPC with a compressive strength of 190 MPa was selected. The aggregate has a maximum grain size of 0.5 mm, the steel fibers have a length of 6 mm. The composition is given in Table 2. 

**Figure 3:**
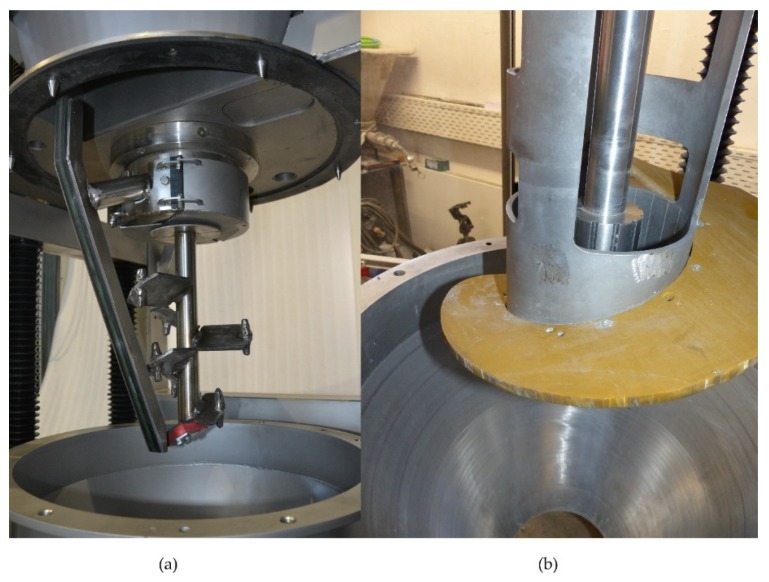
(**a**) Mixing with inner and outer agitator of the KNIELE KKM-RT; (**b**) Coaxial-system with attached spiral for measuring in absolute units; high 170 mm; radius inner cylinder 38 mm and inner radius outer cylinder 85 mm.

The production of the mortar and the rheological measurements were performed in a single machine. The raw materials of the UHPC were mixed in the KNIELE KKM-RT with the mixing tools (Figure 3a) at an agitator speed of 3 m/s for 3 min. The mixing tools with quick-change device were then removed and the measuring device (Figure 3b) installed. The inner cylinder has a height of 17 cm and a radius of 3.8 cm. The outer cylinder has a radius of 8.5 cm.

This unique laboratory mixer with integrated rheometer has a special technical feature compared to other well-known concrete rheometers and has already been described in detail in [12,13]. Conventional coaxial rheometers must implement the pre-shear and the actual measurement with the measuring tool. This inevitably leads to shear-induced particle migration during pre-shearing. In contrast, the pre-shear of the Kniele KKM-RT is implemented by a spiral, which is mounted on the outside of the outer cylinder and counteracts the vertical and the radial separation. The actual measurement is performed by the inner cylinder, which is driven by a synchronous motor. The spiral causes a homogenization of the mixture during the pre-shearing to reduce the thixotropy. The measurement via the inner cylinder is therefore always carried out in a homogeneous material, if the pre-shearing was carried out, previously. With classical rheometers, the measurement is performed in a material which is progressively separated and therefore does not reflect the real conditions. With the experimental setup for the rheometer ICAR a separation in pre-shear and measurement with two separate tools is possible for the first time to avoid segregation during pre-shear. 

In detail: The spiral (Figure 3b) of the KNIELE KKM-RT was attached to the outer cylinder of the coaxial measuring system. When this spiral rotates, the material to be pre-sheared is transported vertically from bottom to top between the outer cylinder and the wall of the mixing vessel. The material passes through openings in the outer cylinder into the shear gap, where it flows downwards. This ensures a three-dimensional movement of the material that actively counteracts the shear-induced particle migration (Figure 3b). In this article, two different measuring regimes are compared. In order to ensure identical initial conditions, the sample was pre-sheared with the rotating spiral for 30 s.

In the traditional measuring regime, the sample was then pre-sheared with the inner cylinder at 1 rev/s for 7 s. The measurement data were not further considered. Then the actual measurement was carried out at the specified speeds for 5 s each.

With the new measuring regime, the measurements were also carried out at the specified speeds. However, before each speed change, the material was pre-sheared with the rotating spiral for 30 s and thus homogenized. The inner measuring tool was stopped during the pre-shearing process. 

## 3. Results

### 3.1. Experimental Setup for the ICAR Rheometer

The raw data of the ICAR rheometer measurements are given in Table 3. These were transferred with the Excel solver according to Reiner-Riwlin (Equations (4) and (5)) into the Bingham and the modified Bingham model.

### 3.2. Results for the Fiber Reinforced UHPC (Mortar) in the Laboratory Mixer KNIELE KKM-RT

The values given in the Table 4 are the mean values of the torques in the period of 1–3 s after the respective speed change. The calculation of the Bingham model parameters is based on the well-established Reiner-Riwlin equation (Equation (4)).

## 4. Discussion

During the use of the ICAR rheometer over several years, segregation could be visually detected after completion of the measurement. After emptying the container, a peripheral zone with a significantly higher content of coarse aggregate often remained in the container. These observations correspond with the pictures in the publication [10]. Thereupon the experimental setup for the rheometer ICAR was developed, which decouples pre-shearing and measurement. 

With the experimental setup for the rheometer ICAR, several assumptions could be verified by measured values. Since TEST 1 and TEST 2 deliver almost identical results, the replacement of the original closed profiled container by the 20 threaded rods used seems to be permitted. Since homogenization in all experiments is performed by the rotating bucket and the whisk, the pre-shearing by the rheometer was reduced to the minimum value of one second in TEST 3 and TEST 4. In TEST 3, the initial torque measured was significantly higher than in TEST 2, and since the pre-shear time with the rheometer was 19 s shorter in TEST 3, segregation due to shear-induced particle migration is likely to have been significantly lower. The required torques for the individual speed stages were therefore higher. However, since shear-induced particle migration also takes place in TEST 3, the values in the lower speed range are closer to TEST 2. To ensure that homogeneous material is initially present in each speed stage, in TEST 4 the test set-up was homogenized before each speed change. The torques measured in the individual speed stages are significantly higher than in the previous measuring regimes. It is noticeable that the greatest differences are found especially at the low speeds. In order to better classify the measured values of TEST 4, TEST 5 was carried out with the measuring regime from TEST 3 afterwards. TEST 4 was carried out with a significant time lag from the previous tests, so that hydration processes or the well-known workability loss of the superplasticizer [14] should be taken into account. In addition, the mix was intensively homogenized seven times in TEST 4. Negative effects of this long and intensive mixing (overmixing) on the workability are also conceivable. [15] described that the mixing quality cannot be significantly increased by repeated and prolonged mixing. However, the particle collisions lead to particle abrasion and thus to an accumulation of fines in the mixture. The abrasion of early hydration products is also mentioned. As a result, the surface area is noticeably increased, thus increasing the demand for water and superplasticizer. The consequence is a deterioration of the flow properties. However, the motor whisk requires a minimum speed to ensure circulation within the rotating bucket. There is a tendency to work for too long and at too high speed, so that an overmixing effect must be taken into account.

In order to counteract the workability loss and overmixing, the following procedures should be implemented:

- Limiting the fresh concrete age during rheological measurements in order to minimize superimposed hydration effects.
- Use of binders that ensure stable fresh concrete properties over long periods of time.
- Use of superplasticizers with extended consistence retention or addition of a separate consistence retaining admixture to maintain stable fresh concrete properties over long periods of time.
- At present, the stand mixer and the motor whisk are operated manually. Due to the automation of the processes with a programmable logic controller (PLC), which is currently being implemented, the measuring time will be considerably reduced.
- The danger of over-mixing will be reduced by shortening the pre-shearing time and adjusting the speed of the whisk. The manual speed control of the motor whisk is also automated by a PLC.

The noticeably higher measured values in TEST 5 compared to TEST 3 suggest that TESTS 1–3 and 4,5 should be evaluated separately. Since TEST 5 was carried out immediately after TEST 4, the values should be on the safe side. If the rheological properties should have worsened in the short time window, the values of TEST 5 should have tended to increase. However, it can be seen that significant differences in the measured values become apparent due to the changed measuring regime. The calculation of the Bingham and the modified Bingham model parameters based on the Reiner-Riwlin equation prove significant differences in yield stress and for the speed necessary to guarantee full flow in the gap (Table 3). Especially when using the modified Bingham model, the effects of the different pre-shear regimes on the yield stress become apparent. The experimental set-up for the ICAR rheometer proved to be basically suitable. 

In [4], it is assumed for the transformation of the raw data into absolute values that the flow in the shear gap is purely circular. This assumption is not correct for a blade agitator according to [5]. Consequently, a systematic error must be expected when using the ICAR rheometer.

In order to avoid these problems, the tests were carried out on the fiber-reinforced UHPC with the KNIELE KKM-RT. This mixer with integrated rheometer forces a three-dimensional circulation through the spiral with simultaneous homogenization. Even with this fine-grain mortar with a maximum grain size of only 0.5 mm, there are noticeable differences when homogenizing in steps before changing the speed. This high performance mortar with a very high content of superplasticizer shows differences in plastic viscosity. In further investigations it is necessary to clarify what influence the amount of superplasticizer and the addition of fibers have on the rheological parameters yield stress and plastic viscosity. With fiber-reinforced UHPC, it must also be clarified whether the fiber orientation is influenced by the different pre-shearing. 

## 5. Conclusions

The following conclusions could be drawn from the performed investigations:

- An experimental setup for a coaxial rheometer was developed, which allows the pre-shearing of the material without the measuring tool of the rheometer.
- If the pre-shearing is done by the experimental setup, the pre-shearing of the material by the rheometer can be reduced to the minimum specified by the manufacturer. This increases the raw data of the measurements.
- If the material is homogenized by the experimental setup before each speed change of the rheometer, the measured raw data increase further.
- If the raw data of the rheometer are transformed into absolute values with the help of the Reiner–Riwlin equation, there are considerable differences due to the different pre-shear regimes.
- The modified Bingham model provides yield stresses well above zero for the SCC under investigation and therefore appears to reflect the properties better than the classic Bingham model.

The results of this study are important for all applications based on rheological models. It is important to choose a model that can represent the fresh concrete properties as well as possible. In addition, the measured raw data of the rheometer must be determined in compliance with the boundary conditions for rheological measurements. In [4], the authors refer, among other things, to the maintenance of homogeneity during the entire measurement period.

## 6. Patents

The laboratory mixer is covered by a European patent with the designation EP3181216A1.

## Figures and Tables

**Table 1:** Composition of the self-compacting concrete (SCC) investigated.

Material	[kg/m^3^]
cement (CEM) III/A 52.5 N	280
limestone powder	250
superplasticizer	2.5
water	140
rhine gravel 8/16	452
rhine gravel 2/8	617
rhine gravel 0/2	669

**Table 2:** Composition of the ultra-high performance concrete (UHPC) investigated.

Material	[kg/m^3^]
CEM I 52.5 N	857
fly ash	104
superplasticizer	35
water	242
basalt sand 0.5 mm	830
silica	222
polypropylene fibers	3
steel fibers	77

**Table 3:** Results for the comparative measurements of the ICAR rheometer under different boundary conditions. Carried out with a SCC according to Table 1.

Parameters	TEST 1	TEST 2	TEST 3	TEST 4	TEST 5
measurement	with ring	without ring	without ring	without ring	without ring
	continously	continously	continously	individual steps	continously
pre-shear time ICAR blade agitator [s]	20	20	1	1	20
time after water addition [min]	30	33	36	60	70
**rev/s**	**Torque [Nm]**				
0.5	1.13	1.186	1.507	1.828	1.602
0.425	0.894	0.993	1.088	1.549	1.322
0.35	0.757	0.8	0.882	1.364	1.1
0.28	0.578	0.622	0.666	1.135	0.902
0.2	0.427	0.474	0.549	1.018	0.667
0.125	0.279	0.311	0.324	0.786	0.454
0.05	0.102	0.119	0.128	0.497	0.215
**Bingham-Model**					
yield stress [Pa]	0	0.3	0	66	10.1
plastic viscosity [Pa·s]	43.3	46.5	54.5	54.1	59.9
min. full flow speed [rev/s]	0	0.001	0	0.25	0.033
**Modified Bingham-Model**					
yield stress [Pa]	3	4.1	14.3	74.7	12.9
plastic viscosity [Pa·s]	36.6	40.6	26.7	44.7	54.9
c [Pa·s^2^]	3.36	2.48	14.7	3.52	2.033

**Table 4:** Raw data from the measuring and calculated values based on the Reiner-Riwlin equation for the UHPC.

Speed	New Regime	Traditional Regime
rev/s	Nm	Nm
1	0.839	0.599
0.8333	0.693	0.55
0.6666	0.602	0.472
0.5	0.454	0.338
0.3333	0.336	0.236
0.1666	0.147	0.177
0.0833	0.185	0.118
0.0166	0.0825	0.092
yield stress [Pa]	25.6	25.1
plastic viscosity [Pa·s]	31.5	22.8
minimal full flow speed [rev/s]	0.157	0.213
